# Acute psychosocial stress and working memory performance: the potential of physical activity to modulate cognitive functions in children

**DOI:** 10.1186/s12887-019-1637-x

**Published:** 2019-08-05

**Authors:** Kathrin Wunsch, Maria Meier, Lea Ueberholz, Jana Strahler, Nadine Kasten

**Affiliations:** 1 Institute of Sports and Sports Science, Karlsruhe Institute of Technology, Engler-Bunte-Ring 15, Building 40.40, 76131 Karlsruhe, Germany; 2 Department of Sport Science, University of Freiburg, Freiburg, Germany; 3 Department of Psychology, University of Konstanz, Konstanz, Germany; 4 Department of Safety and Quality Regulations, University of Wuppertal, Wuppertal, Germany; 5 Department of Psychotherapy and Systems Neuroscience, Justus Liebig University Giessen, Giessen, Germany; 6 Department of Psychology, University of Trier, Trier, Germany

**Keywords:** Stress-buffering effect, Cross-stressor adaption hypothesis, Working memory, Trier social stress test for children (TSST-C), Ecological momentary assessment

## Abstract

**Background:**

Research suggests that physical activity (PA) enhances cognitive performance and prevents stress-related impairments of higher order cognitive functions like working memory (WM) performance. The aim of the current study was to investigate the effect of PA on WM performance after acute stress exposure in preadolescent children.

**Methods:**

Regular PA was assessed for seven consecutive days during a typical school week using accelerometers in a sample of 44 preadolescent children (14 girls, *M*_age_ = 11.29 years, *SD*_*age*_ = 0.67). Following this period, participants performed an automated operational span (OSPAN) task immediately after being exposed to the Trier Social Stress Test for Children (TSST-C).

**Results:**

Children exhibited prototypical response slopes in salivary cortisol and salivary α-amylase as markers of the endocrine and autonomic stress response immediately after psychosocial stress induction. A subsequent two-way ANOVA comparing high- and low-stress responders revealed a significant interaction between group affiliation and PA level on WM performance for both stress markers. Interestingly, best WM performance was demonstrated in children showing both high PA levels and high cortisol (or low α-amylase, respectively) stress responses.

**Conclusions:**

Though patterns differed for salivary cortisol and salivary α-amylase, overall findings suggest that PA buffers the negative effects of stress on cognitive performance in children.

## Introduction

Children face multiple stressful situations in their everyday lives, including homework [1], standardized testing situations, and presentations [2]. Importantly, children are required to cognitively perform at their full potential within these stressful situations at school. Especially in times when it is most critical to perform at their best, the desire to do so and the resulting stress impairs performance [3]. As a key aspect of cognitive functioning, working memory (WM) is the concept responsible for the transient holding and manipulation of information to regulate thoughts and behavior [4]. In adults, cognitive performance (i.e. WM) at high work-loads [5] and in complex tasks [6–8] is negatively affected by stress [9].

Though far less investigated in children [10, 11], results revealed a negative influence of psychosocial stress on complex WM performance during childhood [10]. However, Quesada and colleagues did not find an effect of acute psychosocial stress on WM performance in two simple (instead of complex) span tasks [11], mirroring evidence in adult populations [12].

These negative effects of stress on cognitive performance are supposed to be modulated by stress-related activity of the hypothalamic pituitary adrenal (HPA) axis, as high amounts of glucocorticoid receptors can be found in areas associated with WM, such as the prefrontal cortex [6, 13–15]. Consequently, aspects of WM relying on prefrontal cortex function are negatively influenced by increased levels of glucocorticoids during acute stress [8]. Taken together, results point towards WM impairments caused by cortisol-related effects of psychosocial stress, especially if WM task demands are high [5]. Regular engagement in physical activity (PA) may be a promising approach to encounter these repercussions as PA is found to attenuate these detrimental effects of cortisol on WM performance.

PA is associated with numerous health benefits in adults and (school-aged) children (see [16, 17], for reviews) and buffers deleterious effects of stress on health (stress-buffer hypothesis; [18, 19]). The stress-buffering effect of PA is proposed to be a promising mechanism to prevent stress related complaints and diseases [19, 20]. The cross-stressor adaptation (CSA) hypothesis [21, 22] provides a possible biological explanation for this effect. It assumes that PA elicits unspecific adaptations of the physiological stress system (comprising the autonomic nervous system (ANS) and the HPA axis; i.e. a habituation), which may cause a reduced sensitivity to subsequent homotypic (e.g. physical) and heterotypic (e.g. psychosocial) stressors [22, 23]. Whilst there is good evidence for attenuated responses of (habitually) active individuals to homotypic stressors, evidence is diverse for heterotypic ones [19, 24–27]. More recent investigations are inconclusive, with some providing no evidence for the CSA hypothesis [28, 29], whereas others (at least partly) support the CSA hypothesis for different physiological parameters [26, 30–34]. So far, studies examining these coherences in children mainly focused on stress responses of the ANS [35–37], commonly measured by means of cardiovascular parameters. Here, findings reliably show attenuating effects of PA on ANS responses. To the best of our knowledge, only one study investigating the CSA hypothesis in children assessed endocrine stress markers of the HPA axis [38]. In this study, findings indicated a reduced endocrine stress response to an acute psychosocial stressor in children with higher amounts of PA. Apparently, there has not been a study examining salivary biomarkers of ANS responses in children until today. However, as salivary α-amylase (sAA) is known to reliably elicit immediate reactions to acute stress [39], this biomarker should be considered as an alternative sympathetic stress marker in upcoming investigations. Studies examining the CSA hypothesis in children and considering both stress axes concomitantly are still pending. Given the stress axes’ varying responsiveness to similar stressors and different response trajectories (the fast response of the ANS and the delayed response of the HPA axis) (see e.g., [40]), disparate links with WM performance are to be expected. More studies are needed to examine PA as a buffering agent for stress-related health outcomes and to investigate underlying mechanisms of this buffering effect, especially in children. Taken together, results are inconclusive in adults, and results of studies focusing on children point to attenuated ANS and HPA response patterns in more active subjects.

Research indicates that regular engagement in PA is able to not only protect from stress related health complaints, but to also improve cognitive functions (e.g. WM) in children and adolescents [41–43]. Especially children might benefit from PA due to, e.g., their high capability for neural plasticity and rapid adaptability of neuroendocrine functions [42, 44, 45]. A study by Koutsandreou, Wegner, Niermann and Budde [46] replicated findings of earlier studies on effects of chronic exercise on WM performance in children (e.g. [43, 47, 48]) and revealed that WM performance significantly increased in school children aged 9 to 10 years following a 10-week exercise intervention. These results were confirmed by two more recent studies, first of which showed that an 8-week intervention of 20 min exercise per day during school time elicited benefits for WM performance [49]. Another study on acute exercise effects revealed improvements in inhibitory control and information-processing elicited by a single session of 20 min of intermittent exercise [50]. Interestingly, the beneficial effects of an acute (coordinative) exercise session on cognitive performance (i.e. attentional performance) in school children have been shown to be related to neuronal connections between the cerebellum and the prefrontal cortex [51]. When considering the opposite direction of this relationship, studies revealed no impact of cognitive fatigue on physical performance [52].

To date, numerous studies revealed a positive relation between regular PA or exercise and performance in different cognitive tasks in children, especially for cognitive control and WM performance [46, 47, 53, 54]. As mentioned above, PA positively modulates brain functions and structures, as well as behavioral aspects of cognition [55]. In their everyday lives, children regularly face situations in which they find themselves under pressure when solving highly demanding cognitive tasks. Research has shown a negative influence of perceived pressure (i.e. stress) on WM performance [11], but concurrently indicated beneficial effects of PA on these cognitive functions [56] and has shown that PA is able to prevent stress related complaints and diseases [19, 20] when carried out on a regular basis. However, nothing is known about the potential stress-buffering effect of PA on cognitive performance. Therefore, aim of the current study was to examine whether impairing effects of acute stress on a highly demanding cognitive task are less pronounced in children with high habitual PA levels compared to their low active counterparts. Consequently, the first objective was to (A) expand upon evidence for the CSA hypothesis in children by examining potential effects of PA on stress responses of the ANS and HPA system measured by salivary biomarkers. The second objective (B) was to explore if higher amounts of PA in children can protect cognitive capacities from negative effects of stress. It was assumed that active participants show (A) attenuated stress reactions and (B) advanced cognitive performance in stressful situations as compared to their low active counterparts.

## Methods

### Participants

Fifty-five children (21 girls, *M*_age_ = 10.82 years, *SD*_*a*ge_ = 0.72) were recruited at secondary schools in Freiburg, Germany, with sample size being comparable to similar studies (e.g. [7, 11, 57]). Children were either recruited via newspaper announcements or their schools were contacted for recruitment and testing permission. Participants were derived from different types of secondary schools (e.g. higher secondary education (“Gymnasium”), middle secondary education (“Real−/Gesamtschule”) and lower secondary education (“Waldorfschule”)). Whereas most studies on biological stress markers only focus on male participants as the menstrual cycle of females is known to strongly influence those parameters, the current study included both sexes, but excluded females who already reached puberty [58]. Additionally, participants were excluded if they were younger than 10 or older than 12 years to control for age related differences in salivary biomarkers [59]. Children were also excluded if they suffered from any neurological or psychological disease or reported regular medication intake. Prior to testing, legal guardians and participating children gave their written informed consent. With this consent form, legal guardians completed the eligibility screening, where they were asked questions regarding above mentioned exclusion criteria and some demographic questions. Participants did not receive any financial compensation. Eleven children had to be excluded from the following analyses because of invalid PA data (see below). Accordingly, the final sample consisted of 44 preadolescent children (14 girls, *M*_age_ = 11.29 years, *SD*_*age*_ = 0.67).

### Procedure

**Fig. 1:**
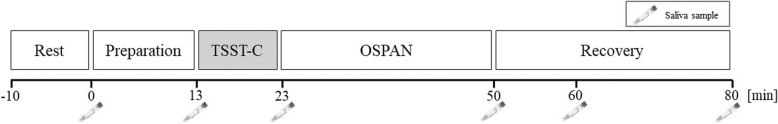
Overview of the study procedure for the laboratory session. TSST-C = Trier Social Stress Test for Children. OSPAN = automated operation span task

The current study is of observational nature including both, cross-sectional (across all children) and longitudinal (repeated measurements for stress responses) analyses. All procedures were in accordance with the Declaration of Helsinki and the study’s design and procedures were approved by the ethics committee of the University of Freiburg (AZ: 254/16). The study consisted of two assessments, with the first objectively measuring participant’s habitual PA using accelerometry and ecological momentary assessment over seven consecutive days in a typical school week. Following this one-week ambulatory assessment period, children were scheduled for the second, laboratory examination to assess their stress reactivity as well as their WM performance. Each child was tested individually and all sessions started between 1 and 3 p.m. to control for circadian variations in salivary biomarkers (e.g. [60]). Additionally, children were asked to refrain from eating and drinking sugar-containing beverages for 2 hours prior to and to rinse their mouth with tab water immediately before the testing session to avoid artificially heightened levels of salivary biomarkers. The detailed study procedure for the laboratory session is depicted in Fig. 1.

After arriving at the preparation room, children were welcomed by the experimenter and were given a short resting period of 10 min to reduce anticipatory heightened stress levels and to make them feel comfortable. Afterwards, participants underwent the child version of the Trier Social Stress Test (TSST-C; [61]) in a separate room. In between there was a 3-min period for changing rooms and giving last instructions in the TSST-C-room. The TSST-C is a common standardized method to experimentally induce psychosocial stress. It has been proven to elicit both ANS and HPA axis responses [62] and has been evaluated repeatedly (e.g. [63, 64]). All children were naive to the applied stress procedure. The TSST-C comprised a 10-min preparation period followed by a 5-min free speech and a 5-min mental arithmetic task performed in front of a committee. In the free speech part children were asked to complete a story, the beginning of which was told by the experimenter. Children were instructed to continue this story for 5 minutes in a most exciting way.

Following the TSST-C, WM performance was assessed using an automated operation span task (OSPAN; [65, 66]) back in the preparation room. After completion of the OSPAN, participants remained seated for another 30 min to examine recovery of salivary biomarkers. The entire testing session lasted approximately 90 min.

All participants completed the study design in the designated way. As the focus of the current study does not inherently rely on the influence of stress on WM but rather on the influence of PA on WM performance under stressful constraints, a no-stress control group was not included. However, cognitive performance was controlled for by measuring intelligence in a non-stressful condition prior to testing.

### Measurements

#### Physical activity

Analogous to previous studies [67, 68] PA data was collected for seven consecutive days in ordinary school weeks, using a direct triaxial accelerometry-based motion sensor (AiperMotion 440, Aipermon GmbH, Munich, Germany), which has been shown to gain reliable data [69, 70]. The motion sensor automatically analyzes the data with disclosed online algorithms classifying activity into “rest”, “low active”, “moderately active” and “high active” (in minutes). These categories were pooled over a day to receive the total amount of moderate-to-vigorous intensity physical activity (MVPA) per day. This amount was then summarized over all days with valid wear-time registration and was then divided by days with sufficient wear-time registration to receive a mean time of MVPA per day. Children were requested to wear the accelerometer during waking hours on a belt on the side of their non-dominant hip and to only remove it for sleeping, water activities (i.e. showering or swimming) or in case of acute injury risk (i.e. contact sports). They were excluded from analysis if they did not wear the accelerometer on at least 4 days with a minimum of 8 h wear-time registration per day. As reported above, eleven children had to be excluded based on this criterion.

Concomitantly to activity recording, children received a smartphone for ecological momentary assessment (EMA). Using movisensXS, Version 0.8.4211 (movisens GmbH, Karlsruhe, Germany), children received questions about their PA twice a day (1 and 7 p.m.), asking about activities done and their perceived intensity on a scale of 0 (not exhausting at all) to 10 (very exhausting). Based on these specifications, accelerometer data was screened for non-wearing times and was complemented by EMA data if necessary.

Based on the global recommendations of the World Health Organization [71], children were labelled to be physically active if they exhibited at least 60 min of MVPA per day. Based on this, 11 children (seven girls) in our data set were classified as active. The remaining children exhibited an average of less than 60 min of PA per day and were therefore classified as low active.

#### Stress response

Salivary α-amylase (sAA) and salivary cortisol (sCort) were used as biological indicators of children’s stress response to the TSST-C. sAA is known to be an indicator for ANS activity [72], whereas sCort release is an indicator for HPA activity in response to an acute stressor, especially when psychosocial stress is induced by a performance task containing socio-evaluative threat and uncontrollability [63]. Both markers have been shown to be valid alternatives that are easily and non-invasively collected, without a need for specific training or equipment, and they do not generate additional stress like blood sampling which is known to cause falsely positive results [73]. Saliva samples were obtained via an absorbent device (Salivette® Cortisol; Sarstedt, Numbrecht, Germany) at six assessment points: 0, 13, 23, 50, 60, and 80 min with reference to the end of the resting period (see Fig. 1 for an overview of sampling points). Saliva samples were collected by instructing the children to keep the swab in their mouth for 1 minute and roll the swab around, but to not chew. Samples were stored at − 20 °C immediately after testing and were sent to Dresden Lab-Service GmbH (Germany) for biochemical cortisol analysis, where they were thawed and spun at 3.000 rpm for 3 min to obtain clear saliva. Free cortisol concentrations (nmol/l) were determined by a luminescence immunoassay for the in vitro diagnostic quantitative determination of cortisol in human saliva (IBL International). Samples were immediately re-frozen after determination and were then sent to the biochemical laboratory of the Department of Clinical Biopsychology in Marburg. After thawing and re-centrifuging, sAA activity was measured using a kinetic colorimetric test and reagents obtained from Roche (Roche Diagnostics, Mannheim, Germany). Saliva was diluted 1:625 using 0.9% saline solution. The reagents contained oligosaccharides (here 4,6-ethylidene-(G7) p-nitrophenyl-(G1)-α, D-maltoheptaoside), which are cleaved into fragments by α-amylase. Fragments are further hydrolyzed by an α-glucosidase to yield p-nitrophenol. The rate of formation of p-nitrophenol is directly proportional to the samples’ amylase activity and was detected using an absorbance reader at 405 nm (Spectrostar nano, BMG Labtech, Ortenberg, Germany). Inter- and intra-assay coefficients of variation were below 8.5% for both determinations.

There were no biologically implausible values for both biological parameters. sCort exhibited a negligible amount of missing data points (i.e. less than 1%). For sAA, however, there was a larger proportion of missing values, particularly due to insufficient amount of saliva. Therefore, seven participants had to be excluded from the following sAA analyses as less than 50% of their saliva samples were valid.

#### Working memory performance

As mentioned above, WM performance was used as an indicator of cognitive performance in children and was examined by means of a modified version of the automated operation span task (OSPAN; [65, 66]) as done before in a study examining the association of fitness to WM performance in children [74]. Stimuli were presented focally on a 10.1 in. Windows tablet (i.onik, Paderborn, Germany) using the Psychology Experiment Building Language [75]. Within the OSPAN, simple arithmetic distractor tasks (processing tasks) were combined with a set of target letters which had to be remembered for later recall (storage task; [66]). As soon as an arithmetic task such as “3 + 4 – 5 =? ” was presented on the screen, participants were asked to solve the task as fast as possible and to touch the tablet screen to indicate they calculated the result. Then, a single digit (e.g. “5”) appeared, as well as a “correct” and a “false” button to indicate the presented digit as being the correct or false result to the arithmetic task. Subsequently, a target letter was presented for 1000 ms [74], which children were instructed to remember. After three to seven items (with the number of items per trial varying randomly to avoid that participants anticipate the number of letters to be recalled), 12 letters were presented in a 3 × 4 matrix and participants had to recall the letters presented during the last trial in correct serial order by clicking on the appropriate letters. This untimed recall screen marked the end of a trial and was followed by a feedback screen indicating the number of correct answers for 1000 ms before the next trial started immediately.

OSPAN scores were calculated by summing the total number of correctly recalled letters (i.e. partial-credit unit scoring, see [76]). As research suggests that stress impairs WM performance only at high loads [6], only trials with six or seven items were considered for the subsequent analyses. Additionally, an accuracy criterion was set at 50% [74]. No child had to be excluded based on this criterion.

#### Covariates

##### Demographics

Demographic information about sex, age and stage of pubescence was collected prior to examination via a questionnaire completed by legal guardians of children.

##### Body-Mass-Index

Children’s body weight (in kg) and height (in cm) were retrieved within the questionnaire. The body-mass-index (BMI) was calculated as body weight (in kg) divided by height squared (cm^2^).

##### Intelligence

In order to (a) avoid learning effects of rehearsed OSPAN completion and (b) keep the temporal effort for children at a minimum, a measurement of cognitive performance in a non-stressful setup was included. To compare baseline levels regarding cognitive performance, children completed the Raven’s Standard Progressive Matrices Test (SPM; [77, 78]) which is considered a measure of abstract reasoning [66] and has strong relationships to the concept of fluid [79] and general [80] intelligence. The SPM consists of five subsets (A to E) with 12 items each that progressively get more difficult and was administered as a self- paced power test. Participant’s total amount of correct answers was transformed into T- values [77].

#### Statistical analyses

A multilevel growth curve approach using the lme4 package [81] in R version 3.4.3. was applied to analyze changes in the two salivary biomarkers over time, as this approach allows for concurrent estimation of both, within-subject trajectories on level 1 and interindividual differences on level 2 [82].

Since no study exists until today examining the three-factorial relationship between physical activity, stress and cognitive performance, previous studies on bivariate relationships did not provide information regarding the size of anticipated effects in multilevel models. Moreover, as the present study had to deal with substantial sample-size constraints due to limited budget, no a priori power analysis but a minimum detectable effect size (MDE) approach was implemented [83]. This approach can be used to indicate the standardized effect size that could be detected with an appropriate level of power given a specific sample size at both levels. Overall, small direct effects of level-1 can be detected in the current design as well as large cross-level interaction effects given a power of 80%.

## Results

Since no experimental manipulation of PA but a quasi-experimental classification of children was adopted, it is important to ensure that groups are comparable regarding important characteristics. Table 1 displays participant characteristics separated by low active and active children. The two groups are comparable regarding age, BMI and intellectual capacity. However, there was a significant difference in sex with girls being more active than boys.

**Table 1:** Participant characteristics separated by low active and active children

	Low active group	Active group	Comparison	
n	33 (75%)	11 (25%)		
Age	11.33 (± 0.65)	11.19 (± 0.74)	*t*(42) = 0.58,	*p* = .56
Sex			*χ*^*2*^(1) = 6.84,	*p* = .02
Male	26 (87%)	4 (13%)		
Female	7 (50%)	7 (50%)		
BMI^a^	17.25 (± 2.33)	16.22 (± 2.18)	*t*(36) = 1.22,	*p* = .23
SPM	42.79 (± 5.48)	43.09 (± 6.94)	*t*(42) = −0.15,	*p* = .88
Baseline sCort	4.73 (± 3.37)	4.25 (± 3.07)	*t*(42) = 0.42,	*p* = .68
Baseline sAA^a^	225.82 (± 186.22)	202.01 (± 113.04)	*t*(36) = 0.39,	*p* = .70

*BMI* Body-Mass-Index, *SPM* Standard Progressive Matrices, *sCort* salivary Cortisol, *sAA* salivary α-Amylase

*Note:* ^a^only 38 participants provided valid data

### Biological stress response and PA

Since both biological stress parameters exhibited considerable deviations from normal distribution, data was transformed prior to analyses. With regard to sAA, the log-transformation was applied, whereas sCort data was normalized using Box-Cox power transformation as this procedure has been shown to produce superior results [84]. First, unconditional growth models were set up including both, a linear (i.e. *time*) and a curvilinear (i.e. *time*^*2*^) change over time [82]. Results are presented in Table 2.

**Table 2:** Estimated fixed effects from the unconditional growth model for salivary cortisol (sCort) and salivary α-amylase (sAA)

	sCort		sAA	
	Coefficient	*p*	Coefficient	*p*
Intercept, *π*_0*i*_	1.5530	< .001	5.2300	< .001
time, *π*_1*i*_	0.0412	< .001	0.0007	.837
time^2^, *π*_2*i*_	- 0.0005	< .001	−0.0004	.302

*Note:* time^2^ was modelled as a fixed effect

Regarding sCort, the unconditional growth model indicated a prototypical pattern of change over time, comparable to trajectories observed in other studies on children (e.g. [59, 61]). Here, sCort levels initially increased after stress exposure, reached a peak level at *– π*_*1i*_/(*2* ∙*π*_*2i*_*)* (i.e. at 41 min), and subsequently decreased again. For sAA on the other hand, the unconditional growth model indicated no change over time, as the coefficients associated with *time* and *time*^*2*^ (i.e. *π*_*1i*_ and *π*_*2i*_) failed to reach significance. However, variance components associated with the linear change over time were highly significant for both, sCort (${\sigma}_1^2$ = 0.0003, *p* < .001) and sAA (${\sigma}_1^2$ = 0.00004, *p* < .001), signifying that there is still high interindividual variation in change trajectories. Apparently, some children exhibited high responses after being exposed to psychosocial stress, whereas others showed attenuated responses or did not respond at all. Deducing from the CSA hypothesis, some of this variation should be attributable to differences in children’s PA status. However, the inclusion of PA as a level 2 predictor did not lead to significant differences in baseline values or slopes in the current study. Additionally, neither sex nor age had an effect on trajectories.

To further analyze whether the extent of responses had an impact on WM and how this could be modulated by PA, high- and low-responders for both biological measures were separated by means of a post-hoc median split as suggested by Elzinga & Roelofs [85], based on absolute differences between peak and baseline values for both biomarkers. Interestingly, children who showed high increases in sAA levels after stress exposure did not necessarily exhibit a pronounced sCort peak and vice versa (*χ*^*2*^(1) = 0.67, *p =* .41). Hence, further analyses were carried out separately for the two biological parameters to account for possible differential effects.

**Fig. 2:**
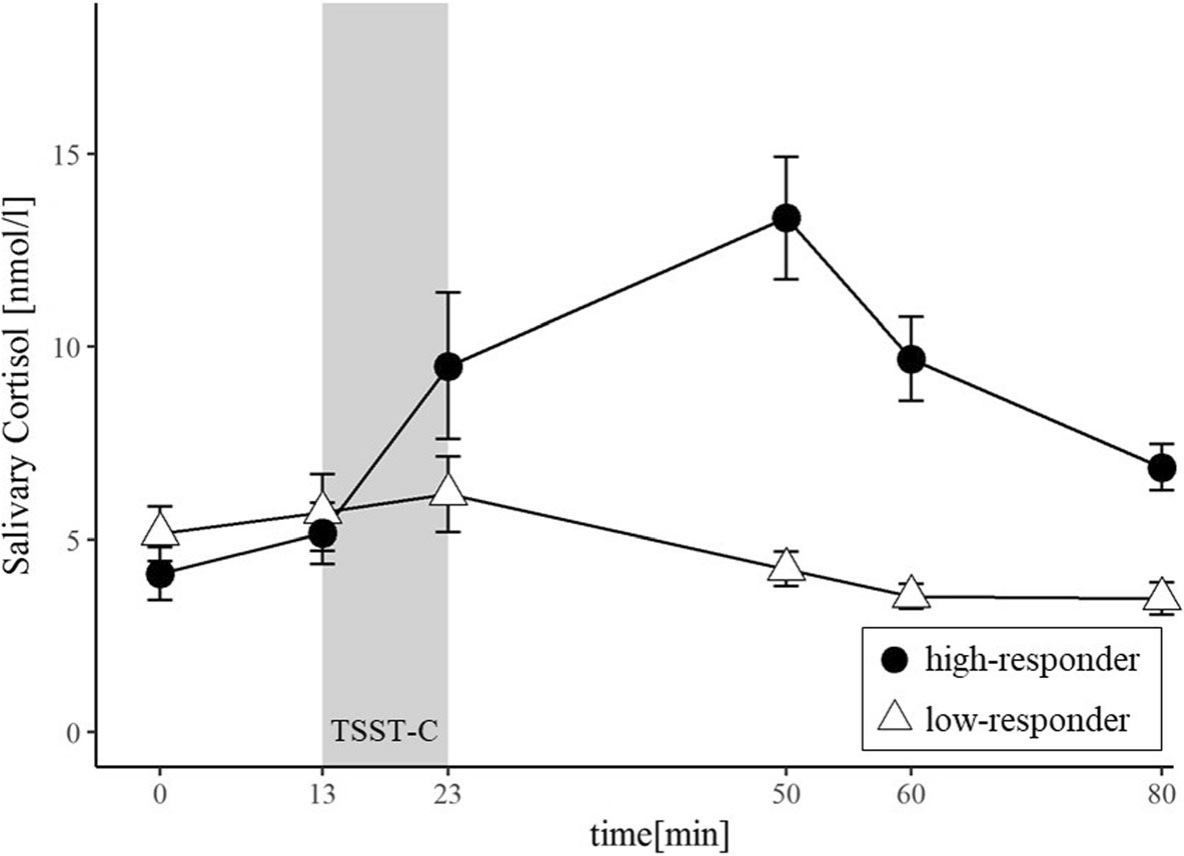
Mean (± SE) salivary cortisol concentrations for high-responders (*n* = 23) and low-responders (*n* = 21) during the laboratory session

**Fig. 3:**
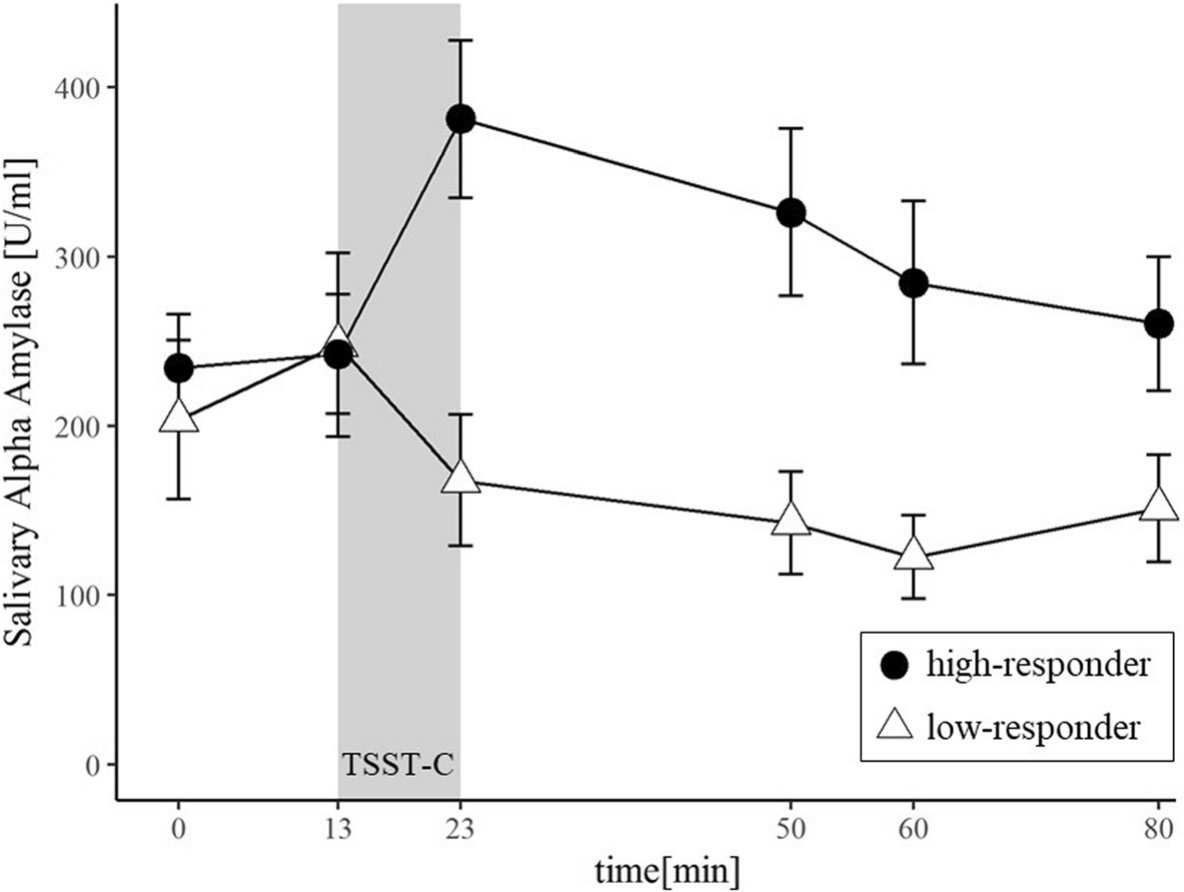
Mean (± SE) salivary α-amylase concentrations for high-responders (*n* = 19) and low-responders (*n* = 18) during the laboratory session

For both, sCort and sAA, high and low-responders were comparable regarding age (sCort: *t* (42) = 0.12, *p* = .91; sAA: *t* (35) = 1.20, *p =* .78) and gender (sCort: *χ*^*2*^(1) = 0.12, *p* = .91; sAA: *χ*^*2*^(1) = 2.57, *p =* .17). Unsurprisingly, inclusion of the group variables as level 2 predictors explained a significant amount of variance in individual change trajectories. More specifically, unexplained variance associated with the linear change over time declined by 41% for sCort and by 22% for sAA. Estimated fixed effects from the conditional growth models are presented in Table 3. Additionally, raw sAA and sCort trajectories for both groups are displayed in Fig. 2 and Fig. 3 respectively.

**Table 3:** Estimated fixed effects from the conditional growth model for salivary cortisol (sCort) and salivary α-amylase (sAA)

Fixed Effects	sCort		sAA	
	Coefficient	*p*	Coefficient	*p*
Intercept, *π*_0*i*_	1.8680	< .001	4.9990	< .001
group	−0.6078	.104	0.2874	.339
time, *π*_1*i*_	0.0018	.794	−0.0134	<.010
group	0.0759	< .001	0.0298	< .001
time^2^, *π*_2*i*_	−0.0001	.132	0.0001	.056
group	−0.0007	< .001	−0.0003	< .001

*sCort* salivary Cortisol, *sAA* salivary α-Amylase

*Note:* time^2^ was modelled as a fixed effect; group was added as a dummy-coded variable with 0 = low-responder and 1 = high-responder

Now, the absent effect for *time* and *time*^*2*^ for sAA within the unconditional model becomes apparent. Indeed, the expected changes over time are evident, but only for children who exhibited a pronounced sAA response after stress exposure. Accordingly, the cross-level interactions *time x group* and *time*^*2*^ *x group* became significant within the conditional growth model (see Table 3).

### Working memory performance

To examine the effect of PA on WM performance after stress exposure, two ANOVAs with WM performance as dependent variable and two between-subject factors were performed: (1) PA status (low active vs. active) and (2) reactivity (high-responder vs. low-responder), with the latter factor being operationalized in terms of sCort and sAA reactivity.

**Fig. 4:**
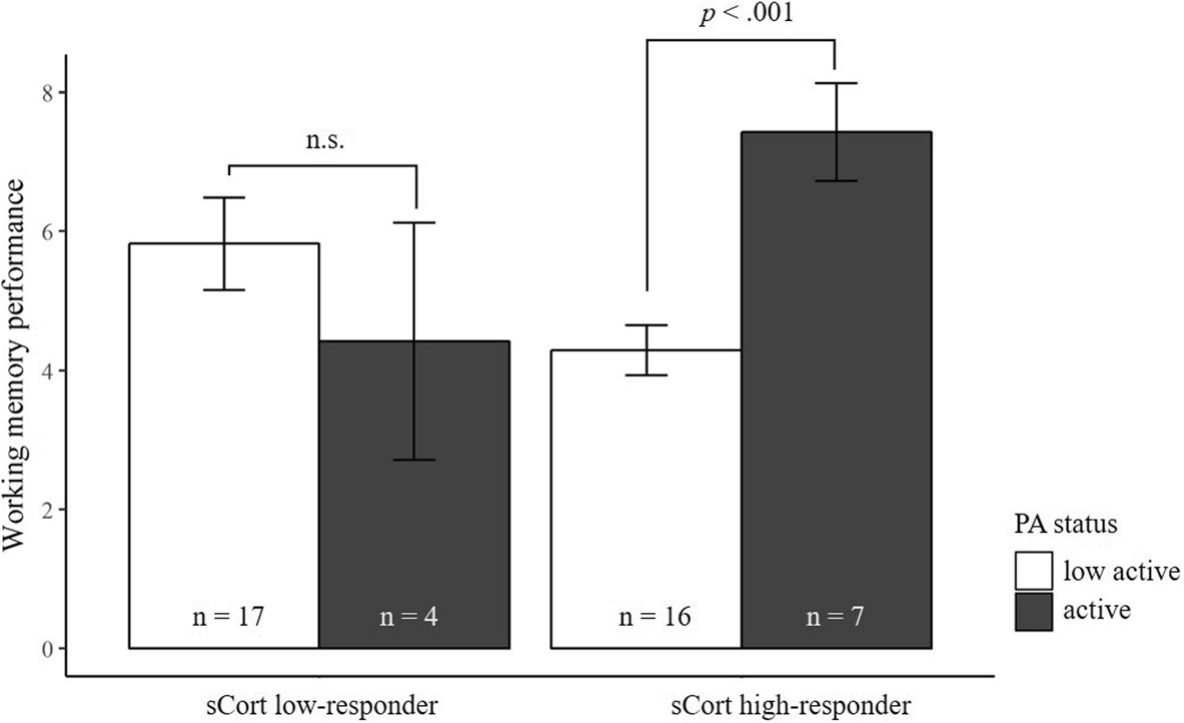
Mean (± SE) working memory performance for salivary cortisol (sCort) high-responders and low-responders divided by physical activity (PA) status

**Fig. 5:**
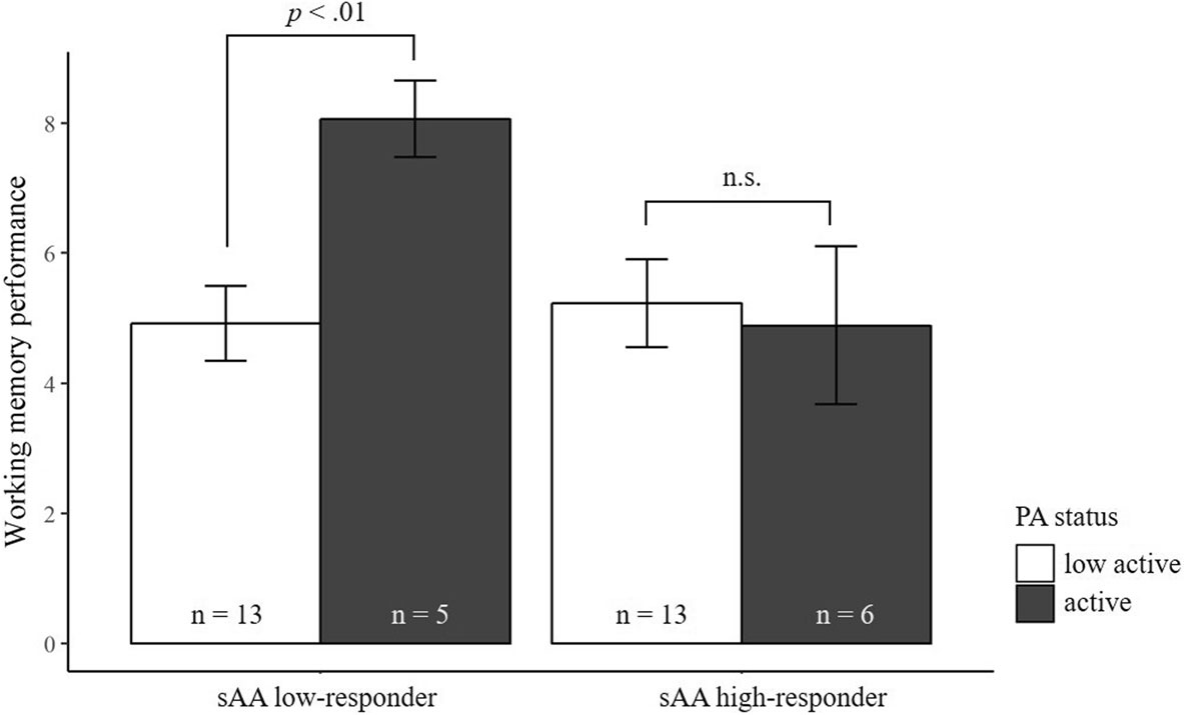
Mean (± SE) working memory performance for salivary α-amylase (sAA) high-responders and low-responders divided by physical activity (PA) status

WM performance was not impaired by stress as there was no main effect for reactivity irrespective of whether group affiliation was based on sCort (*F* (1, 40) = 0.20, *p* = .65, *η*_*p*_^*2*^ = .01) or sAA reactivity (*F* (1, 33) = 0.79, *p* = .38, *η*_*p*_^*2*^ = .02). Similarly, there was no main effect for PA in both ANOVAS (for sCort: *F* (1, 40) = 2.74, *p* = .10, *η*_*p*_^*2*^ = .06; and for sAA: *F* (1, 33) = 2.43, *p* = .13, *η*_*p*_^*2*^ = .07). Even if no main effect reached significance, both ANOVAs exhibited a significant interaction between PA status and stress reactivity (for sCort: *F* (1, 40) = 7.77, *p* < .01, *η*_*p*_^*2*^ = .16; for sAA: *F* (1, 33) = 4.42, *p* < .05, *η*_*p*_^*2*^ = .12), indicating there are indeed beneficial effects of PA (see Fig. 4 and Fig. 5). Neither the inclusion of sex nor age showed any impact on these results.

With respect to sCort, post-hoc t-tests indicated that there was no difference between active and low active children when sCort concentration was low (*t* (19) = 0.89, *p* = .39, d = 0.41). However, when concentration increased after psychosocial stress induction (i.e. in the group of sCort high-responders), there was a large difference between activity groups regarding WM performance. Specifically, active children exhibited superior performance in the OSPAN task compared to low active children (*t* (21) = − 4.38, *p* < .001, d = 1.99). It is to accentuate that the former group (i.e. active and high cortisol responses) exhibited higher WM performance scores than the other subgroups combined (*t* (42) = − 2.52, *p* < .05, d = 1.29).

When classification into high- and low-responders was based on sAA increase after stress induction, a different pattern appeared. There was no difference in WM performance between the two activity groups within high-responders (*t* (21) = − 4.38, *p* < .001, d = 0.13). Among low-responders, however, active children showed significantly elevated WM performance compared to low active children (*t* (16) = − 3.09, *p* < .01, d = 1.63). Again, the former group (i.e. active and low sAA responses) exhibited higher WM performance scores than the other subgroups combined (*t* (35) = − 2.81, *p* < .01, d = 1.03).

## Discussion

### Main findings

The present study aimed to investigate potential beneficial mechanisms of PA in children that enable them to attain their best cognitive performance in stressful situations. The first objective (A) was to expand upon evidence for the CSA hypothesis in children by examining potential effects of PA on stress responses of the ANS and HPA system. The second objective (B) was to explore if higher amounts of PA in children can protect cognitive capacities from negative effects of stress. Based on previous studies it was assumed, that active participants show attenuated stress reactions and advanced cognitive performance in stressful situations as compared to their low active counterparts. Multilevel growth curve analyses and ANOVAs were applied and revealed that (A) higher amounts of PA were not associated with an attenuated physiological stress response, that (B) PA had a positive effect regarding sCort on WM performance in children. Furthermore, the two stress systems, ANS and HPA, responded intraindividually independent. As such, sCort high-responders did not necessarily also reveal a high sAA response.

### Cross-stressor adaptation

The assumption that active children show an attenuated physiological stress response as compared to low active children (as proposed by the CSA hypothesis) was not supported. PA showed no effect on the trajectory of children’s stress responses for either biological parameter. Partly, this is contrary to former investigations showing that heart-rate responses as an indicator of ANS activity are attenuated in children showing higher amounts of PA [35–37]. Although sAA responses were repeatedly shown to be associated with ANS responses to stress in children and adolescents [86–88], higher amounts of PA were not related to an attenuated sAA response to stress in the current study. Hence, the assumption about an association between PA and ANS stress responses derived by studies measuring heart-rate could not be confirmed. However, studies employing sAA as autonomic stress marker are sparse. The few available studies on adults are in accordance with the present null finding [29, 34, 89]. Effects of PA on sAA stress reactivity in children have not yet been investigated.

The finding of no relation between attenuated ANS responses and PA status was paralleled by the result that the endocrine stress response measured by sCort was not blunted in active as compared to low active children. In adult populations, evidence is inconclusive. Some studies examining endocrine stress responses in adults showed physical fitness or high PA to have an attenuating effect on sCort concentration following a laboratory stressor [30–33, 90, 91], whereas others failed to find a significant effect [28, 29, 92] or did not find any difference in sCort responses [93]. Until today, only one study investigated the relationship between objectively measured PA and biological reactions to a laboratory stressor in children [38]. Although the findings of this study support the CSA hypothesis, our results did not replicate these effects.

It is worth noting that differences in age might play a crucial role within child populations. While Martikainen and colleagues [38] studied 8-year old children, the current sample was on average 3 years older. Although children who already reached puberty were excluded, this exclusion was based on self-report data. Hence, the two populations may not be inherently comparable what could account for inconsistencies in findings. It is possible that factors such as sleep, social support, nutrition or higher experience in scholastic presentations are more relevant to biological responses in children between 10 and 12 years and thus override the attenuating effects of PA. Besides the difference in age, the approach of classifying children into activity groups substantially differed in former studies. While Martikainen and colleagues [38] used terciles, classification in the present study was based on global recommendations of the World Health Organization [71]. Thus, children were labelled to be physically active if they exhibited at least 60 min of MVPA per day. Albeit, only 25% of children fulfilled this guideline. Yet, it is still worth noting that some studies point towards the fact that the biological plausibility of the CSA hypothesis has not been supported by research on exercise and exercise-related adaptations [19, 94]. Given the small sample size in the current study, the non-supportive effects have to be interpreted with caution and future studies with greater sample size and higher statistical power are needed to disentangle the complex interactions of PA and endocrine or autonomic stress reactivity in children of different age.

### Stress, physical activity and working memory performance

Second aim of this study was to investigate whether PA exerts a beneficial effect on WM performance in stressful situations. Current results revealed that PA indeed offered a benefit in children with a low ANS response to psychosocial stress, as well as in children with a distinct HPA response. Thus, children who exhibited lower levels of sAA after the TSST-C exhibited superior performance in the WM task if they were physically active. In contrast to the ANS response, children did not benefit from a higher amount of PA if they exhibited a low HPA response, but rather when they showed a distinct response. This implies that both stress systems have different impacts on WM performance. The response of the ANS seems to rather prevent the beneficial effects of PA on WM, i.e. there was no effect of PA status in sAA high-responders. sCort findings appeared completely different. Here, effects of PA only appeared in children showing a high HPA response. As the two stress systems show distinct temporal trajectories, these differences can possibly account for the present findings. However, it can only be speculated upon the possible differences in effects the two stress systems cause on WM in distinct temporal proximity to the stressor. As the peak of the major agents of the ANS and HPA are temporally distinct in reference to stressor cessation, it is possible that the systems exert their effects on WM at different time points during the WM task independently from each other [95].

### Methodological considerations

Importantly, the current design took the two major methodological limitations of existing studies on implications of stress on WM performance in adults (as well as in children) into account. First, the temporal course of the physiological stress response was neglected in former studies [5] and as a result, there was a lack of temporal proximity of WM assessment and stress experience (e.g. [96]); second, the limited complexity of the WM task was considered (e.g. [85, 97–99]). Precisely, WM performance appears to be no longer impaired by stress 35 min after stressor cessation [85]. The endocrine stress response peaks approximately 10 to 20 min after stressor cessation [62]. Possibly, this offers an explanation why no impairing effect of stress on WM was found in studies in which WM was assessed 20 min after cessation of the stressor at the earliest. When WM is assessed immediately after stress exposure, however, impairments were found more reliably [6, 7]. This was taken into account while the current design was compiled. In conclusion, timing matters when stress effects on WM are investigated and thus, the differences in designs could explain the inconclusive findings so far [95]. Additionally, it is still possible that the individual motivation and dedication to perform well in high demanding cognitive tasks plays a critical role in testing situations [100] and therefore should be controlled for in future studies.

Developmental differences might explain the absence of a negative effect of stress on WM performance in some studies, even when the methodological limitations mentioned above are taken into consideration (e.g. [11]). Studies in the field of developmental neuroscience provide evidence for age-dependent variations in stress sensitivity from infancy to adolescence [101, 102]. While infants do hardly respond to social stress, stress sensitivity (as indicated by an increase in biological stress markers following stress exposure) increases during childhood and adolescence with adult-like responses in late adolescence [103, 104]. Besides this impact of chronological age, puberty is a major contributor to stress sensitivity as well. Given previous reports, one might cautiously assume higher sensitivity to social stress with higher pubertal development ([64, 105], for a recent review of both factors see [58]). Hence, both age and pubertal development need to be taken into account when examining sensitivity to stress. However, such developmental changes in cognitive sensitivity to stress received little attention until today. The hippocampus, amygdala, and prefrontal cortex for instance are not fully developed during childhood (for review see [45, 106]) and the density of stress hormone receptors in the prefrontal cortex of children is lower than in adolescents or adults [107, 108]. Consequently, a child’s brain might be less sensitive to stress (i.e. due to smaller amounts of receptors or transmitters, or a different receptor sensitivity). Therefore, cognitive impairments could, for example, only be present following high levels of stress or prolonged stress situations [102]. Interestingly, in a study on young rodents, spatial WM impairments were only observed after a longer duration of corticosterone treatment, but not after a shorter period [109]. This might imply even larger WM impairments in children suffering from chronic or prolonged stress. Future studies will have to tell whether any beneficial effect of PA also applies in this case.

### Critical reflection of the study design

Besides above mentioned methodological strengths, there are multiple other strengths of this study worth mentioning. (1) A standardized and valid stress protocol (TSST-C) was applied that created a stress situation which strongly resembles situations children encounter on a daily basis at school (i.e. speaking in front of the class) and is therefore highly relevant. (2) Biological markers of the endocrine and autonomic stress response systems were evaluated simultaneously in the present study which provides a more comprehensive picture of the acute biological stress response. (3) PA was assessed objectively through direct accelerometry of sufficient duration to be representable of children’s daily activity and through an EMA at the same time. (4) WM performance was measured by a stress-sensitive, complex WM task with high task-demands, thereby ensuring reliable assessment of stress-induced task interference. (5) The time interval between stress exposure and WM assessment was kept at a minimum to measure immediate stress effects of stress on WM performance.

The listed strengths of the current study extenuate many limitations of previous research. Certain limitations of the present study merit discussion though. First of all, the PA data assessment was not without difficulties. The average daily wearing time of the accelerometer ranged from 3 h 11 min to 13 h 26 min a day with significant differences in the average activity levels between short wearing times and long wearing times (*p* > .05). To enhance the validity of the estimation, the data of children who wore the accelerometer for less than 8 h per day on at least 4 days was excluded. Another challenge of collecting objective activity data through direct accelerometry is that participants removed the accelerometer (at least) whilst participating in contact or water sports. However, this data is of particular importance when assessing habitual PA. This was accounted for in the present study by replacement of missing accelerometer data with EMA data. However, EMA data is highly subjective and relies on the children’s information about their daily PA. It is obvious that this kind of information is vulnerable to bias. The merging of direct and indirect PA assessment is without doubt an improvement to one-method assessments and is recommended for future studies aiming to measure habitual PA in children. Regardless, self-reported PA scores may present an index of motivation rather than actual PA level and may affect the quality of data. Motivational reinforcements for both, the objective and subjective PA assessment should be considered to increase validity of data. Further it has to be taken into account that PA and physical fitness are two distinct constructs that correlate only moderately with each other [110]. Studies investigating the CSA hypothesis in children merely focused on acute bouts of exercise or PA [35–37]. Possibly, a high amount of PA is still not sufficient enough to provoke adaptations of the physiological systems in the same manner as physical fitness is known to do in respect to homotypic stressors. Hence, future studies should aim at objectively measuring physical fitness additionally to PA to deliver a deeper understanding of this relationship.

Another limitation of the present study is that interference of causal pathways is only speculative due to the observational design [111]. It is therefore imperative to conduct experimental studies to validate findings and indicate causality. It is of great importance to examine different PA and exercise interventions in children, ideally utilizing follow-up periods at the cessation of the program to indicate whether benefits are maintained.

The final, general limitation discussed here is the restricted sample composition and sample size. Although effect sizes indicate moderate differences between low active and active children, power might be insufficient due to small sample sizes. Accordingly, post-hoc power analyses using G*Power [112] confirmed this assumption with regard to the analyses of the relationship between PA and WM performance. Though both ANOVAs indicated a medium-sized main effect for PA (i.e. *η*_*p*_^*2*^ = .06 for sCort and *η*_*p*_^*2*^ = .07 for sAA), power was rather small (1-*β* > .40). To reach an appropriate power, however, sample size needs to be twice as big as in the current study. Even if other studies on similar topics (e.g. [85]) only examined half of the participants, a duplication of sample size would be favourable. Furthermore, the voluntary participation and recruitment strategies might have introduced a sampling bias. Another shortcoming which needs to be mentioned is that children’s school affiliation was not recorded, rendering it impossible to control for school-specific differences in children. Ignoring this additional clustering of the data beyond the nesting of measurement points within children might have led to biased standard error estimates [113]. Additionally, the generalizability of this study is limited to healthy adolescents who have not yet reached puberty. Further, when interpreting the results, it should be noted that both dependent variables, WM and the stress response, are complex processes, which can be influenced by many factors.

## Conclusion

The current levels of stress and PA in children support the relevance of further investigations on those variables in children. Free-time activities have been reduced in children whereas stress levels increased [114]. During school time, physical education classes are strictly limited to a very few hours per week [115], falling far below the recommended 60 min of MVPA for children per day [116]. Whilst the risk of a sedentary lifestyle for children’s physical health are better understood, only little is known about the complex direct and indirect effects of PA on cognition in children. Early interventions seem to be particularly important, as especially during childhood and early adulthood, systems linked to cognitive outcomes like the prefrontal cortex still form and can be modified [42, 45].

For certain, more randomized-controlled trials and experimental, longitudinal studies including several measurement points, thus not only accounting for stress response measurement, but also for ontogenetic development of these reaction across a larger period of time, depicting time-dependent variation regarding sensory-motor development and puberty-related changes of children and adolescents are needed to understand causal effects of lifestyle factors like PA on stress and cognition. Also, brain imaging studies have the potential to help to understand the proposed stress-buffering mechanisms of PA [117]. In a first approximation, the present results suggest that PA is able to diminish the negative effects of stress on cognitive performance in children. With respect to biological mechanisms, best WM performance was demonstrated in children showing higher PA levels and high stress-induced cortisol or low α-amylase, respectively. As both systems, the HPA axis and the ANS, are essentially involved in the adaptive response to acute stress, findings of opposing links with WM are counterintuitive at first sight. However, the systems vary in their degree of responding to the same stressor and they show different time trajectories in responding. Different effective directions are thus not entirely surprising and future studies will have to examine the partially parallel but rather complementary effects of HPA and ANS reactivity (see also the discussion on the stress coherence/compensation model; [118]). These results can help to discover the role of PA in both, the development of cognitive functions and the direct and indirect enhancement of children’s cognitive performance through an increased stress resilience. Obtained insights are of particular importance for the development of future recommendations regarding intensity, frequency and duration of daily periods of PA among children and adolescents to prevent decreases in cognitive performance due to acute stress.

## Data Availability

The datasets used and/or analysed during the current study are available from the corresponding author on reasonable request.

## Abbreviations

ANS: Autonomic nervous system
CSA: Cross-stressor adaption
HPA: Hypothalamus pituitary adrenal
MVPA: Moderate-to-vigorous intensity physical activity
OSPAN: Automated operational span task
PA: Physical activity
sAA: Salivary α-Amylase
sCort: Salivary cortisol
TSST-C: Trier Social Stress Test for Children
WM: Working memory
